# Biomarkers of Glucose Metabolism Alterations and the Onset of Metabolic Syndrome in Survivors of Childhood Acute Lymphoblastic Leukemia

**DOI:** 10.3390/ijms23073712

**Published:** 2022-03-28

**Authors:** Katarzyna Konończuk, Katarzyna Muszyńska-Rosłan, Karolina Konstantynowicz-Nowicka, Maryna Krawczuk-Rybak, Adrian Chabowski, Eryk Latoch

**Affiliations:** 1 Department of Pediatric Oncology and Hematology, Medical University of Bialystok, 15-274 Białystok, Poland; kmroslan@post.pl (K.M.-R.); maryna.krawczuk-rybak@umb.edu.pl (M.K.-R.); 2 Department of Physiology, Medical University of Bialystok, 15-222 Białystok, Poland; karolina.konstantynowicz@umb.edu.pl (K.K.-N.); adrian@umb.edu.pl (A.C.)

**Keywords:** ALL, childhood cancer survivors, CCS, children, diabetes biomarkers, obesity, overweight, C-peptide, ghrelin, gastric inhibitory peptide (GIP), glucagon, insulin, plasminogen activator inhibitor-1 (PAI-1), resistin, leptin, visfatin

## Abstract

Owing to advances in treatment modalities and supportive care, overall survival rates have reached up to 90% among children with acute lymphoblastic leukemia (ALL). However, due to the underlying illness and therapy, they are at a greater risk of developing lifestyle diseases. Hence, special attention is paid to early detection of the components of metabolic syndrome (MetS). This study aimed at investigating the association of plasma levels of nine diabetes markers with being overweight and components of MetS in ALL survivors. The study included 56 subjects with mean age of 12.36 ± 5.15 years. The commercially available Bio-Plex Pro Human Diabetes 10-Plex Panel kit was used to evaluate levels of diabetes biomarkers. ALL survivors presented statistically higher concentrations of GIP (*p* = 0.026), glucagon (*p* = 0.001), leptin (*p* = 0.022), and PAI-1 (*p* = 0.047), whereas the concentration of ghrelin was lower (*p* < 0.001) compared to the control group. Moreover, subjects within normal BMI range showed higher GIP (*p* = 0.005) and lower ghrelin concentration (*p* < 0.001) compared to healthy peers. At least one risk factor of MetS was present in 58.9% of participants, who showed significantly higher levels of C-peptide (*p* = 0.028), leptin (*p* = 0.003), and PAI-1 (*p* = 0.034) than survivors who did not meet any MetS criteria. In conclusion, ALL survivors are at greater risk of disturbances in carbohydrate metabolism. Understanding the pathogenesis and applicability of diabetes markers is crucial for developing strategies to prevent metabolic syndrome in ALL survivors.

## 1. Introduction

The most common malignancy diagnosed in childhood is acute lymphoblastic leukemia (ALL). Due to remarkable advances in treatment modalities and excellent supportive care, overall survival rates have reached up to 90% or more depending on the risk group [1]. As a result, the population of adolescents and young adults who have experienced childhood cancer therapy is growing significantly. Nowadays, the world is witnessing an increase in lifestyle diseases leading to cardiovascular incidents that can result in premature death. Recent studies also indicate an enhanced risk of earlier onset of age-related chronic diseases in childhood cancer survivors (CCS). The main causes of increased morbidity and mortality among CCS include obesity, insulin resistance (IR), hypertension (HT), dyslipidemia, diabetes mellitus (DM), and consequently metabolic syndrome (MetS) and cardiovascular disease [2,3].

The best-studied detrimental factors in the development of metabolic diseases in ALL survivors at a younger age include the use of glucocorticosteroids and cranial radiotherapy (CRT) during treatment, yet not all agents have been identified so far. The etiopathogenesis of the earlier occurrence of the many diseases among CCS has not been clearly elucidated. Some studies suggest that triggers may include chemotherapeutics used in childhood that alter carbohydrate and lipid metabolism (anthracycline and asparaginase, among others) [4,5]. In addition, poor eating habits and reduced activity persisting beyond the completion of treatment enhance the risk of obesity and lifestyle diseases in this population [6,7].

The metabolic pathways are regulated by the number of factors whose changed secretion may be associated with the onset of insulin resistance and obesity [8]. The major markers include C-peptide, ghrelin, gastric inhibitory peptide (GIP), glucagon, insulin, plasminogen activator inhibitor-1 (PAI-1), resistin, leptin, and visfatin. The detailed characteristics and roles in metabolism are shown in Table 1. As the number of survivors increases substantially, there is a growing interest in the early detection of the predisposition to develop late effects in this particular population. 

This study aimed at investigating the association of plasma level of nine diabetes markers with being overweight, insulin resistance, and components of metabolic syndrome in childhood survivors of acute lymphoblastic leukemia.

## 2. Results

The clinical characteristic of ALL childhood survivors is presented in Table 2. The mean age at diagnosis was 5.01 ± 3.46 years, and follow-up after cessation of the treatment was 6.58 ± 4.64 years. The age and sex did not differ between the study and control groups. 

Compared to the study group, the control group presented statistically higher concentrations of GIP (*p* = 0.026), glucagon (*p* = 0.001), leptin (*p* = 0.022), and PAI-1 (*p* = 0.047), whereas the concentration of ghrelin was lower (*p* < 0.001). We did not find any differences in the C-peptide (*p* = 0.386), insulin (*p* = 0.158), resistin (*p* = 0.429), and visfatin (*p* = 0.066) levels (Table 3).

In the analysis stratified by body mass index (BMI) value, ALL survivors with normal BMI range demonstrated greater levels of GIP (4993.09 ± 10750.06 pg/mL vs. 487.86 ± 278.73 pg/mL, *p* = 0.005) and lower levels of ghrelin (296.64 ± 232.40 pg/mL vs. 764.97 ± 557.20 pg/mL, *p* < 0.001) when compared to the control group. In turn, overweight and obese survivors presented higher levels of glucagon (572.58 ± 327.20 pg/mL vs. 363.39 ± 230.83 pg/mL, *p* = 0.006) and leptin (9180.43 ± 6989.29 pg/mL vs. 4952.19 ± 4642.43 pg/mL, *p* = 0.034) than normal weight subjects. Subsequently, we performed an analysis comparing survivors with a division by BMI value and control group (normal BMI survivors vs. overweight and obese survivors vs. control group). As before, the analysis revealed an increased GIP level among survivors with normal BMI (*p* = 0.029), whereas leptin and glucagon levels were higher in the overweight and obese subgroup (*p* = 0.009, *p* < 0.001, respectively). Finally, decreased ghrelin level was found in both normal BMI and high BMI survivors in comparison to the control group (*p* < 0.001). 

According to sex, C-peptide (*p* = 0.014), leptin (*p* = 0.031), and resistin (*p* = 0.028) levels were higher in females than males within the study group. Moreover, ALL females showed higher insulin (*p* = 0.041) and leptin (*p* = 0.003) levels, while ALL males had higher levels of GIP (*p* = 0.036) compared to the control group.

We also assessed the diabetes markers by the time of cessation of anticancer treatment. The participants over 5 years after the end of treatment presented higher levels of PAI-1 (5524.84 ± 2338.48 pg/mL vs. 3711.59 ± 2360.43 pg/mL, *p* < 0.001) and resistin (12247.92 ± 7238.51 pg/mL vs. 5182.93 ± 3620.10 pg/mL, *p* = 0.002) than subjects with a shorter time after completion of treatment. 

In the further analysis, we checked the relationship between diabetes markers and risk factors of metabolic syndrome. At least one risk factor was present in 58.9% of the participants. Twenty-nine subjects had increased waist circumference (WC), eleven met the criterion related to blood pressure, six had elevated glucose levels, three presented decreased HDL levels, and six had elevated triglycerides (TG) levels. Compared to the control group, survivors with MetS factors showed greater levels of GIP (*p* = 0.030), glucagon (*p* < 0.001), leptin (*p* = 0.001), PAI-1 (*p* = 0.009), and lower level of ghrelin (*p* < 0.001) (Table 4). 

ALL survivors with two or more MetS risk factors compared to the control group presented higher levels of C-peptide (*p* = 0.008), glucagon (*p* < 0.001), insulin (*p* = 0.020), leptin (*p* < 0.001), PAI-1 (*p* = 0.001), and a lower level of ghrelin (*p* = 0.001) (Table 4). 

Compared to survivors who did not meet any MetS criteria, the ALL survivors with at least one MetS risk factor presented significantly higher levels of C-peptide (*p* = 0.028), leptin (*p* = 0.003), and PAI-1 (*p* = 0.034). In addition, we investigated the subset of participants who met two or more MetS risk factors in comparison to those without any risk factors. Beyond higher C-peptide (*p* = 0.002), leptin (*p* < 0.001), and PAI-1 (*p* = 0.031) higher insulin levels (*p* = 0.021) were found. The results of the CCS analysis with and without the presence of metabolic derangements are presented in Table 5.

The subgroup of ALL survivors meeting the metabolic criterion for a diagnosis of insulin resistance (10.7%) showed higher levels of C-peptide (1150.13 ± 626.88 pg/mL vs. 425.84 ± 528.95 pg/mL, *p* = 0.005), glucagon (625.22 ± 355.41 pg/mL vs. 284.69 ± 177.93 pg/mL, *p* = 0.042), and leptin (9930.65 ± 6458.18 pg/mL vs. 2906.16 ± 4843.62 pg/mL, *p* = 0.016) in comparison to subjects with normal HOMA-IR. 

Furthermore, a strong positive correlation was found between BMI and C-peptide (*r* = 0.56, *p* < 0.001), glucagon (*r* = 0.52, *p* = 0.001), and leptin (*r* = 0.56, *p* <0.001). A positive correlation between PAI-1 and cholesterol levels (*r* = 0.53, *p* = 0.041) was observed. Leptin was strongly associated with HOMA-IR (*r* = 0.72, *p* = 0.002). In addition, a strong correlation between PAI-1 and resistin (*r* = 0.83, *p* < 0.001) was found.

There were no differences in the levels of any analyzed biomarkers in subjects treated with both radiotherapy (RT) and hematopoietic stem cell transplantation (HSCT). 

**Figure 1:**
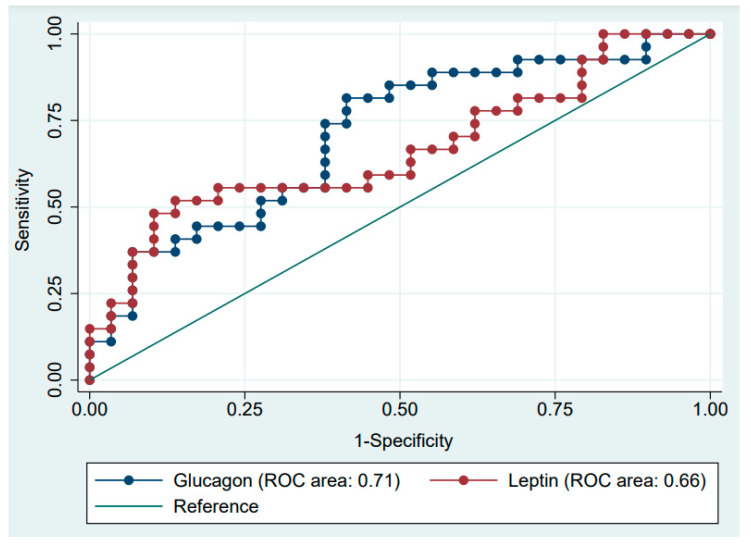
The receiver operating characteristic (ROC) analysis for prediction of overweight/obesity based on the serum levels of glucagon and leptin in childhood acute lymphoblastic leukemia survivors.

The receiver operating curve (ROC) analyses were conducted to assess the diagnostic profile of diabetes markers with the presence of any metabolic syndrome features (Table 6) and overweight/obesity in the study group. Glucagon (AUC 0.71, *p* = 0.003) and leptin (AUC 0.67, *p* = 0.026) were found to be predictors for being overweight or obese in ALL survivors; however, we did not observe statistical differences between the two areas under the curve (*p* > 0.05) Figure 1.

Univariate analysis showed a significant association between the presence of at least one MetS risk factor among ALL survivors and levels of leptin (coef. 0.001, *p* = 0.001), PAI-1 (coef. 0.001, *p* = 0.049), and resistin (coef. 0.001, *p* = 0.049); however, the coefficient values were very small. Multivariable analysis confirmed only the effect on leptin level (coef. 0.001, *p* = 0.011).

## 3. Discussion

In this study, we focused on evaluating glucose metabolism biomarkers and their applicability in assessing the late effects associated with the development of metabolic syndrome after childhood cancer treatment. 

Of the nine markers tested, we showed that levels of GIP, glucagon, leptin, and PAI-1 were significantly higher in ALL survivors, whilst ghrelin was lower compared to healthy controls. In addition, our study confirms alterations in carbohydrate metabolism among patients with normal BMI, which may contribute to the onset of metabolic disease in later life.

Due to the greater risk of developing lifestyle diseases in adolescents and young adults after childhood cancer treatment, diagnostic markers are being sought to detect the onset of late complications. There has been an increasing amount of literature focusing on being overweight or obese in childhood as well as more generally in adulthood. In CCS, metabolic syndrome is among the most frequently reported medical conditions that may confer essential public health issues. In this regard, special attention is paid to early detection of the components of metabolic syndrome that promote the development of lifestyle diseases. To successfully prevent possible complications, it is, however, necessary to understand the underlying mechanisms and their etiopathogenesis [8,17].

This is the first study to concurrently and comprehensively evaluate multiple markers of impaired carbohydrate metabolism in individuals after childhood ALL treatment.

Some of the analyzed hormones are well known and widely useful in clinical practice, including diagnosis and treatment of type 2 DM, which is characterized by insulin resistance, resulting in hyperinsulinemia, and, over time, β-cell failure. Elevated C-peptide levels are observed in IR and early type 2 DM. Moreover, in the study by Cabrera et al., C-peptide is a risk factor for coronary heart disease in a population with normal blood glucose levels, and thus according to the authors may be potentially more valuable as an early predictor of coronary events than impaired fasting glucose levels [18]. In our study, C-peptide was higher in patients who met at least one criterion of metabolic syndrome compared to those who did not meet any of the criteria. Similarly, survivors with abnormal HOMA-IR also presented increased level of C-peptide. We did not find any cardiovascular diseases among participants enrolled in the study; however, the mean age of the study was low. We may only speculate that this group of CCS might have a stronger predisposition to developing metabolic syndrome and perhaps cardiac events later in life. Therefore, regular cardiovascular evaluation in these patients is needed [19].

Although the main alteration in type 2 diabetes is β-cell dysfunction and insulin resistance, there is also increased α-cell activity. The result of these abnormalities is hyperglucagonemia, which has long been acknowledged to contribute to hyperglycemia in patients with diabetes by promoting hepatic glucose production. Overweight and obese subjects presented higher glucagon levels compared with both the normal weight and the control group. Furthermore, individuals with any risk factors for metabolic syndrome also demonstrated statistically significant differences compared to the control group. A similar observation was made by Mannell et al., who reported more elevated glucagon levels in obese adolescents with normal glucose tolerance than in lean adolescents. [20]. All these data suggest that subjects with a BMI above the normal range are more predisposed to carbohydrate dysregulation.

A meta-analysis of 23 studies on the role of GIP in patients with type 2 DM revealed increased GIP secretion in subjects with high BMI after glucose or meal stimulation compared to normal weight patients [21]. In our study, we did not perform fasting oral glucose challenge (except for single subjects with impaired fasting glucose); however, we showed higher GIP levels in the whole study group and in subgroups with at least one risk factor for MetS compared to the control group. Interestingly, ALL survivors with a normal weight revealed greater levels of GIP than the control group. In contrast, we did not confirm this trend in participants with a high BMI. The results do not clearly establish the role of GIP in the development of obesity in survivors; nevertheless, they may indicate an inadequate tissue response in children and young adults with a preserved normal body weight. This may be due to the fact that GIP influences the secretion of adipokines and proinflammatory cytokines, which leads to low-grade inflammation and IR, which in turn may promote obesity and MetS [22,23]. However, the relationship between obesity and increased GIP secretion is not well established and further studies are needed.

Ghrelin has been reported to be decreased in obese children and negatively correlated with BMI [24,25]. Our results are consistent with previous findings, and demonstrated that not only the subset of subjects with abnormal BMI had significantly lower ghrelin levels, but also the subgroup with normal BMI, which may indicate a potential detrimental effect of anticancer treatment administered in childhood on ghrelin secretion later in life. This finding may also support the hypothesis that being overweight increases tissue sensitivity to ghrelin. Moreover, the decreased levels of ghrelin have been also observed in patients reaching criteria for metabolic syndrome when compared to controls.

Leptin is a well-known peptide hormone which is mainly secreted by adipocytes and plays an important role in both energy balance and energy expenditure. Leptin level increases in obesity due to enhanced adipose tissue secretion of adipokine and decreases when body weight is reduced. Our study confirmed positive correlation with BMI and HOMA-IR in pediatric population. Furthermore, females presented with higher leptin concentrations compared to males in the study group, and female survivors had greater leptin levels than females in the control group. This finding confirmed leptin as a good predictor of obesity and MetS features [26,27].

In this study, it has been showed that ALL survivors had higher PAI-1 levels than the control group and was a good predictor of features of MetS in univariate analysis. Previous studies in humans also provide evidence that elevated PAI-1 has a direct effect on the development of insulin resistance and type 2 DM, as well as metabolic syndrome. It is possible that the cause leading to the dysfunction may be considered to be endothelial damage and its consequences [19,28,29,30].

Resistin is a poorly established adipokine in ALL survivors. It is known to promote insulin resistance and may be associated with abdominal fat deposition in the general population. Siviero-Miachion et al. found no association between resistin levels and CRT exposure or BMI in young survivors of ALL [31]. Our study showed no difference in resistin levels between the study and control groups. However, women had higher resistin concentrations than men. Additionally, children more than 5 years from the end of treatment demonstrated higher levels of this adipokine than those with a shorter time from the completion of treatment. As studies have shown a decrease in resistin with age, this may indicate lipid abnormalities in this group of patients.

Some studies, but not all, suggested that visfatin may be a potential predictor of insulin resistance in adults. Furthermore, recent studies have revealed that obese and type 2 DM patients presented higher visfatin levels [32,33]. This protein may play a role in contributing to insulin resistance by indicating the secretion of proinflammatory cytokines [34]. Nevertheless, we did not observe any relationship of visfatin with insulin resistance and features of MetS in ALL survivors.

Our study indicates disturbances in carbohydrate metabolism in ALL survivors, which may affect not only the quality and lifespan of this group of individuals, but also the possibility of earlier diagnosis of treatment-related late sequelae that may lead to the development of metabolic syndromes in the future. Dealing with the late effects of cancer therapy raises significant public health issues and substantial medical costs. From an ethical as well as an economic point of view, a preventive strategy is much more justified. Therefore, it is essential to determine the usefulness of new markers in the early diagnosis of complications in this group of patients, perhaps resulting in earlier implementation of preventive interventions among CCS.

In light of the results obtained in our study and the current knowledge about the increased risk of developing metabolic syndrome in ALL survivors, special attention should be paid to educating parents and children about healthy diet and physical activity after treatment. On the other hand, physicians should provide ongoing health care in the form of regular follow-up visits, including assessment of health status, and carbohydrate and lipid parameters, among others, in order to identify early abnormalities and initiate treatment as early as possible. There is still a need for uniform guidelines to identify the group at highest risk of developing MetS and specialized care for these patients. This challenge has been taken up by an international team of experts who are currently developing harmonized guidelines for diabetes and metabolic syndrome screening in childhood cancer survivors. More information is available at www.ighg.org (accessed on 15 February 2022) [35].

The results obtained should be considered in the context of limitations. The study included a small number of patients in whom total body fat mass was not assessed. Therefore, the analysis between the visceral fat percentage and the diabetes markers was not possible. In addition, we did not include a control group of obese children, which might have increased the value of the findings.

To the best of our knowledge, this is the first study to evaluate a broad panel of diabetes markers in individuals who experienced anticancer treatment in childhood. In addition, our research includes a homogeneous group of ALL survivors, relatively long time after cessation of treatment, and no ethnic diversity.

In conclusion, the current study indicates that ALL survivors present abnormalities in carbohydrate metabolism. Subjects with features of metabolic syndrome had higher levels of C-peptide, GIP, insulin, leptin, PAI-1, and lower levels of ghrelin, whereas overweight and obese individuals had increased levels of GIP, leptin, glucagon, and decreased levels of ghrelin. This study may suggest a role of diabetes biomarkers in the pathogenesis and risk assessment of overweight and metabolic syndrome in acute lymphoblastic leukemia survivors. However, further studies are still required to establish the precise significance in pathogenesis and applicability of diabetes markers in the early diagnosis of metabolic syndrome in acute lymphoblastic leukemia survivors.

## 4. Materials and Methods

### 4.1. Study Population

Survivors of childhood acute lymphoblastic leukemia (56 patients, 30 female), who were treated at the Department of Pediatric Oncology and Hematology of the Medical University of Bialystok, were recruited for the study. All the subjects were in continuous clinical remission and participated in a follow-up visit at the clinic. The treatment was administered according to the applicable protocols for each patient using international protocols (The International Berlin-Frankfurt-Münster Group-I-BFM) approved by Polish Pediatrics Leukemia and Lymphoma Group. The written informed consent was obtained from all the participants or their parents. The study was affirmed by the Ethics Committee of the Medical University of Bialystok in accordance with the Declaration of Helsinki (permission number: APK.002.319.2021).

The mean age at the time of the study was 12.36 ± 5.15 years. The control group consisted of 22 healthy peers (8 females) at the mean age of 11.39 ± 4.25 years, with normal body weight, BMI, and fasting blood glucose.

The medical records were performed to obtain data, including age, sex, and anticancer treatment. During the follow-up visit every patient underwent a clinical examination. Anthropometric traits were collected using standard procedure and rigorously recorded. Body Mass Index was calculated as weight in kilograms divided by height in square meters (kg/m^2^). The study group was separated into overweight and obese groups and a subset with BMI in a normal range based on the OLA/OLAF growth charts BMI for age and sex, in which overweight was defined as BMI values +1 standard deviation (SD), while obesity -as +2 SD [36,37]. The waist-to-height ratio (WHtR) was calculated by dividing waist circumference by height, assuming the norm to be <0.5. Blood pressure was measured using a standardized sphygmomanometer (performed three times at 1–2 min intervals); before the measurement, the participants rested peacefully for 5 min. Hypertension was defined as a mean systolic blood pressure (SBP) and/or diastolic blood pressure (DBP) level ≥ 95th percentile adjusted for age, sex, and height [38]. HOMA-IR was evaluated according to the following formula: serum insulin (uIU/mL) x plasma glucose (mmol/L)/22.5. An abnormal HOMA-IR was considered to be more than 2.86 [39]. Echocardiography was performed to assess shortening fraction (SF) and ejection fraction (EF) by a pediatric cardiology specialist.

The metabolic syndrome and its components in children under 16 were defined by the International Diabetes Federation (IDF) recommendations as WC ≥90th centile, triglycerides ≥150 mg/dL, HDL-cholesterol <40 mg/dL, blood pressure ≥130/85 mmHg, fasting glucose ≥100 mg/dL. Among the participants aged 16 and older, MetS was defined by the IDF adult criteria as WC ≥94 cm for men and WC ≥80 cm for women, triglycerides ≥150 mg/dL, HDL-cholesterol <40 mg/dL for men and <50 mg/dL for women, blood pressure ≥130/85 mmHg, and fasting glucose ≥100 mg/dL [40].

### 4.2. Biochemical Analysis

All the laboratory tests were performed following an eight-hour overnight fast. Venous blood samples were stored at −80 °C. The enzymatic colorimetric method was used to measure biochemical parameters.

The commercially available Bio-Plex Pro Human Diabetes 10-Plex Panel kit (catalogue number 171A7001M, Bio-Rad Laboratories, Hercules, CA, USA) was performed on the serum specimens according to the manufacturers’ instructions. The Bio-Plex system allows the simultaneous determination of 10 diabetes-related biomarkers in each well of a 96-well plate. The assay principle was based on the reaction with an antibody against a specific biomarker which was covalently bound to fluorescently dyed magnetic beads, each with a distinct wavelength specific for the target biomarker. The bead-conjugated antibodies reacted with the biomarker sample of interest. After the series of washes, biotinylated antibodies, optional for different epitopes of the target biomarkers, were added to the reaction. The final complex was made by adding a streptavidin-phycoerythrin (SA-PE) conjugate. A dual-laser, flow-based microplate reader -Bio-Plex 200 Reader (Bio-Rad Laboratories, Hercules, CA, USA)—detected the internal fluorescence of the individually dyed beads and the intensity of the signal on the bead surface. The obtained signal was expressed as median fluorescence intensity (MFI), analyzed, and presented as concentration (pg/mL) by the Bio-Plex Manager Software (Bio-Rad Laboratories, Hercules, CA, USA). The concentration of the biomarker attached to the individual beads was proportional to the MFI of the phycoerythrin signal.

### 4.3. Statistical Analysis

Statistical analysis was performed with STATA v. 12.1 (StatCorp, College Station, TX, USA). Normal distribution was examined using the Shapiro-Wilk test. The data were presented as mean ± SD, or median (Me) and interquartile range (IQR) when appropriate. The Mann-Whitney U test was used to assess the difference between two groups, while the Kruskal-Wallis test was applied to compare more than two groups without normal distribution. Analysis of the correlation between parameters was calculated by the Spearman’s rank correlation coefficient. We used the receiver operating characteristic curve to establish the diagnostic values of the diabetes markers. Multivariate analysis was used to examine the association between diabetes markers and the independent variables which potentially might affect their level. A *p*-value less than 0.05 was defined to be statistically significant.

## Figures and Tables

**Table 1:** Descriptive characteristics of diabetes markers [9,10,11,12,13,14,15,16].

Biomarker	Function
C-peptide	A reliable marker of β-cell function. High levels are associated with macrovascular complications. Low levels are associated with microvascular and nerve function complications.
	
Ghrelin	Secreted by pancreatic islets where it stimulates glucagon release and inhibits insulin secretion. Negatively correlates with the occurrence of insulin resistance and obesity in a population with normal fasting glucose. Lower levels are observed in obese people.
	
Gastric inhibitory peptide (GIP)	Secreted by K cells in the duodenum and upper jejunum. High levels are observed in obesity. Normal to increased levels in subjects with type 2 diabetes mellitus.
	
Glucagon	Secreted by pancreatic α-cells. High levels are observed in obesity. Higher fasting glucagon levels are associated with more severe insulin resistance.
	
Insulin	produced by pancreatic β-cells. Hyperinsulinemia affects the development of obesity by inhibiting lipolysis and promoting lipogenesis. Hyperinsulinemia and insulin resistance promote hypertension and atherosclerosis.
	
Plasminogen activator inhibitor-1 (PAI-1)	a pivotal regulator of fibrinolysis. Higher levels have been reported in subjects with obesity, metabolic syndrome, and type 2 diabetes mellitus.
	
Resistin	Secreted mainly by peripheral blood mononuclear cells and macrophages. High levels are associated with promoting inflammation and may be involved in the pathogenesis of insulin resistance and metabolic syndrome.
	
Leptin	Produced by adipocytes. Involved in the low-grade inflammatory state. Contributes to the secretion of proinflammatory cytokines, which results in the development of insulin resistance and type 2 diabetes mellitus.
	
Visfatin	Secreted mainly by adipocytes and macrophages. Higher levels are observed in obese patients and type 2 diabetes mellitus. Possibly involved in the release of proinflammatory cytokines, contributing to insulin resistance.

**Table 2:** Descriptive characteristics of acute lymphoblastic leukemia survivors.

	Study Group	
	Number (%) ^a^ n = 56	Mean ± SD ^b^
Male	26 (46.4)	
Female	30 (53.6)	
		
Age at diagnosis (years)		5.01 ± 3.46
Age at study (years)		12.36 ± 5.15
Follow-up after treatment (years)		6.58 ± 4.64
		
Chemotherapy		
Methotrexate (cumulative dose (mg/m^2^))		10,321.43 ± 6644.50
Cumulative corticosteroid dose (mg/m^2^) ^c^		3538.05 ± 901.85
Prednisone (cumulative dose (mg/m^2^))		1680.00 ± 0.00
Dexamethasone (cumulative dose (mg/m^2^))		277.32 ± 134.60
Cyclophosphamide (cumulative dose (mg/m^2^))		3957.14 ± 2632.93
Anthracycline (cumulative dose (mg/m^2^))		225.00 ± 45.41
		
Radiotherapy	9 (16.1)	
Cranial radiotherapy (CRT) (cumulative dose (Gy))	8 (14.3)	12.75 ± 2.12
Total body irradiation (TBI)	2 (3.6)	12 ± 0.00
No	47 (83.9)	
		
HSCT	6 (10.7)	
		
Metabolic derangements		
1 Metabolic risk factor	21 (37.5)	
2 Metabolic risk factors	7 (12.5)	
3 Metabolic risk factors	4 (7.1)	
4 Metabolic risk factors	1 (1.8)	

^a^ percent of the total; ^b^ standard deviation (SD); ^c^ calculated as prednisone equivalents; *9 the Gray.

**Table 3:** Comparison of diabetes marker concentrations between the ALL survivors and the control group.

	ALL Survivors n = 56	Control Group n = 22	*p* Value
C-peptide (pg/mL)	611.08 (332.59; 962.57)	479.47 (268.64; 799.61)	0.386
Ghrelin (pg/mL)	224.07 (161.76; 356.32)	634.33 (377.65; 1070.13)	<0.001
GIP (pg/mL)	1050.12 (592.44; 1479.55)	417.67 (280.34; 741.02)	0.026
Glucagon (pg/mL)	394.94 (234.92; 612.39)	237.62 (140.22; 324.11)	0.001
Insulin (pg/mL)	530.18 (296.77; 964.83)	377.87 (140.28; 631.33)	0.158
Leptin (pg/dL)	5219.36 (1329.38; 12551.94)	1846.23 (765.72; 3361.22)	0.022
PAI-1 (pg/mL)	4914.04 (3638.52; 6040.11)	3936.78 (3091.16; 4900.93)	0.047
Resistin (pg/mL)	8448.39 (4983.02; 14698.13)	7420.48 (4239.87; 12889.74)	0.429
Visfatin (pg/mL)	1032.53 (689.50; 2632.74)	133.00 (55.58; 1296.86)	0.066

GIP insulin-dependent insulinotropic polypeptide; PAI-1 plasminogen activator inhibitor-1; IQR interquartile range. Data are given as Median and Interquartile range (IQR).

**Table 4:** Characteristic of acute lymphoblastic leukemia survivors according to number of metabolic risk (MetS) factors compared to the control group.

	≥1 MetS Risk Factor n = 33	Control Group n = 22	*p* Value
C-peptide (pg/mL)	792.42 (444.15; 1046.09)	475.47 (268.64; 799.61)	0.094
Ghrelin (pg/mL)	220.49 (183.58; 351.21)	634.33 (377.65; 1070.13)	<0.001
GIP (pg/mL)	1151.39 (592.44; 1731.89)	417.67 (280.34; 741.02)	0.030
Glucagon (pg/mL)	400.89 (269.81; 608.71)	237.62 (140.22; 324.11)	<0.001
Insulin (pg/dL)	588.46 (365.81; 974.98)	377.87 (140.28; 631.33)	0.071
Leptin (pg/mL)	6999.47 (3347.83; 16,562.53)	1846.23 (765.72; 3361.22)	0.001
PAI-1 (pg/mL)	5305.50 (3814.72; 6898.52)	3936.78 (3091.16; 4900.93)	0.009
Resistin (pg/mL)	10,585.10 (5861.14; 15,774.47)	7420.48 (4239.87; 12,889.74)	0.129
Visfatin (pg/mL)	1285.36 (827.91; 3103.90)	133.00 (55.58; 1296.86)	0.068
	**≥2 MetS risk factor** **n = 12**	**Control Group** **n = 22**	***p*** **Value**
C-peptide (pg/mL)	936.48 (591.03; 1546.72)	475.47 (268.64; 799.61)	0.008
Ghrelin (pg/mL)	216.77 (193.13; 286.35)	634.33 (377.65; 1070.13)	0.001
GIP (pg/mL)	704.05 (438.26; 1403.35)	417.67 (280.34; 741.02)	0.138
Glucagon (pg/mL)	445.20 (355.39; 765.60)	237.62 (140.22; 324.11)	<0.001
Insulin (pg/dL)	967.72 (454.60; 1729.53)	377.87 (140.28; 631.33)	0.020
Leptin (pg/mL)	10,981.25 (5654.11; 16,772.59)	1846.23 (765.72; 3361.22)	<0.001
PAI-1 (pg/mL)	5757.94 (4203.10; 7416.83)	3936.78 (3091.16; 4900.93)	0.001
Resistin (pg/mL)	12,026.93 (5382.83; 16,882.36)	7420.48 (4239.87; 12,889.74)	0.299
Visfatin (pg/mL)	1016.43 (971.42; 1760.47)	133.00 (55.58; 1296.86)	0.240

GIP insulin-dependent insulinotropic polypeptide, PAI-1 plasminogen activator inhibitor-1, IQR interquartile range. Data are given as Median and Interquartile range (IQR).

**Table 5:** Characteristic of acute lymphoblastic leukemia survivors according to number of metabolic risk (MetS) factors.

	≥1 MetS Risk Factor n = 33	No MetS Risk Factors n = 23	*p* Value
C-peptide (pg/mL)	792.42 (444.15; 1046.09)	419.15 (258.64; 727.40)	0.028
Ghrelin (pg/mL)	220.49 (183.58; 351.21)	225.45 (118.91; 360.40)	0.817
GIP (pg/mL)	1151.39 (592.44; 1731.89)	1031.18 (605.88; 1093.86)	0.409
Glucagon (pg/mL)	400.89 (269.81; 608.71)	319.44 (174.91; 616.06)	0.220
Insulin (pg/dL)	588.46 (365.81; 974.98)	386.98 (201.06; 814.23)	0.136
Leptin (pg/mL)	6999.47 (3347.83; 16562.53)	3613.77 (664.69; 6269.79)	0.003
PAI-1 (pg/mL)	5305.50 (3814.72; 6898.52)	4478.74 (3409.32; 5383.39)	0.034
Resistin (pg/mL)	10,585.10 (5861.14; 15774.47)	6608.98 (3902.95; 12059.42)	0.059
Visfatin (pg/mL)	1285.36 (827.91; 3103.90)	729.03 (326.13; 2250.56)	0.253
	**≥2 MetS risk factor** **n = 12**	**No MetS risk factors** **n = 23**	***p*** **value**
C-peptide (pg/mL)	936.48 (591.03; 1546.72)	419.15 (258.64; 727.40)	0.002
Ghrelin (pg/mL)	216.77 (193.13; 286.35)	225.45 (118.91; 360.40)	0.959
GIP (pg/mL)	704.05 (438.26; 1403.35)	1031.18 (605.88; 1093.86)	0.788
Glucagon (pg/mL)	445.20 (355.39; 765.60)	319.44 (174.91; 616.06)	0.263
Insulin (pg/dL)	967.72 (454.60; 1729.53)	386.98 (201.06; 814.23)	0.021
Leptin (pg/mL)	10,981.25 (5654.11; 16772.59)	3613.77 (664.69; 6269.79)	<0.001
PAI-1 (pg/mL)	5757.94 (4203.10; 7416.83)	4478.74 (3409.32; 5383.39)	0.031
Resistin (pg/mL)	12,026.93 (5382.83; 16882.36)	6608.98 (3902.95; 12059.42)	0.176
Visfatin (pg/mL)	1016.43 (971.42; 1760.47)	729.03 (326.13; 2250.56)	0.408

GIP insulin-dependent insulinotropic polypeptide, PAI-1 plasminogen activator inhibitor-1, IQR interquartile range. Data are given as Median and Interquartile range (IQR).

**Table 6:** The receiver operating characteristic (ROC) analysis for prediction of at least one metabolic risk factor based on the diabetes markers levels in childhood acute lymphoblastic leukemia survivors.

	AUC	95% Cl
C-peptide (pg/mL)	0.733	(0.596–0.871)
Ghrelin (pg/mL)	0.473	(0.317–0.630)
GIP (pg/mL)	0.638	(0.377–0.899)
Glucagon (pg/mL)	0.642	(0.490–0.795)
Insulin (pg/dL)	0.675	(0.527–0.823)
Leptin (pg/mL)	0.797	(0.684–0.911)
PAI-1 (pg/mL)	0.685	(0.542–0.828)
Resistin (pg/mL)	0.676	(0.531–0.822)
Visfatin (pg/mL)	0.649	(0.429–0.868)

GIP insulin-dependent insulinotropic polypeptide; PAI-1 plasminogen activator inhibitor-1; IQR interquartile range.

## Data Availability

The data that support the findings of this study are available from the corresponding author upon reasonable request. Some data may not be made available because of privacy or ethical restrictions.
